# The Relative Impact of Climate Change on the Extinction Risk of Tree Species in the Montane Tropical Andes

**DOI:** 10.1371/journal.pone.0131388

**Published:** 2015-07-15

**Authors:** Natalia Tejedor Garavito, Adrian C. Newton, Duncan Golicher, Sara Oldfield

**Affiliations:** 1 Faculty of Science and Technology, Bournemouth University, Poole, United Kingdom; 2 Botanic Garden Conservation International (BGCI), Richmond, Surrey, United Kingdom; University of Delhi, INDIA

## Abstract

There are widespread concerns that anthropogenic climate change will become a major cause of global biodiversity loss. However, the potential impact of climate change on the extinction risk of species remains poorly understood, particularly in comparison to other current threats. The objective of this research was to examine the relative impact of climate change on extinction risk of upper montane tree species in the tropical Andes, an area of high biodiversity value that is particularly vulnerable to climate change impacts. The extinction risk of 129 tree species endemic to the region was evaluated according to the IUCN Red List criteria, both with and without the potential impacts of climate change. Evaluations were supported by development of species distribution models, using three methods (generalized additive models, recursive partitioning, and support vector machines), all of which produced similarly high AUC values when averaged across all species evaluated (0.82, 0.86, and 0.88, respectively). Inclusion of climate change increased the risk of extinction of 18–20% of the tree species evaluated, depending on the climate scenario. The relative impact of climate change was further illustrated by calculating the Red List Index, an indicator that shows changes in the overall extinction risk of sets of species over time. A 15% decline in the Red List Index was obtained when climate change was included in this evaluation. While these results suggest that climate change represents a significant threat to tree species in the tropical Andes, they contradict previous suggestions that climate change will become the most important cause of biodiversity loss in coming decades. Conservation strategies should therefore focus on addressing the multiple threatening processes currently affecting biodiversity, rather than focusing primarily on potential climate change impacts.

## Introduction

There is widespread concern that anthropogenic climate change will have significant negative impacts on global biodiversity. Experimental and modelling evidence suggests that climate change could lead to major changes in the distribution of species and the composition of ecosystems, as a result of shifts in temperature and precipitation [1, 2]. Alteration of species composition could lead to associated changes in ecosystem structure and function, and declines in the provision of ecosystem services to humans [1, 3, 4]. Climate change impacts that have already been observed include distributional, phenological and reproductive changes in a wide range of species, together with impacts on community structure and ecological interactions between species [1, 3, 4, 5, 6].

Particular concern has focused on the impact of climate change on the risk of extinction of species. Specifically, it has been suggested that many species will be unable to migrate or adapt sufficiently rapidly to the expected pace and scale of projected climate change, and will therefore be increasingly vulnerable to extinction [3]. Although few recent species extinctions have been attributed directly to climate change, it has been suggested that climate change could surpass habitat loss as the principal threat to global biodiversity in coming decades [2, 4]. However, assessments of the potential loss of species as a result of climate change remain highly uncertain, partly because of the wide variety of different approaches that have been used by previous analyses [2, 7]. For example, Thomas et al. [8] used projected changes in distributional ranges of species together with species-area curves to estimate changes in species richness. Results suggested that by 2050, 15–37% of species in the regions studied may be “committed to extinction” under intermediate climate warming [8]. Similar approaches were used by Malcolm et al. [9] in their study of biodiversity hotspots, where projected percent extinctions ranged from <1 to 43% of the endemic biota (mean 11.6%), and by van Vuuren et al. [10], who projected a 2–4% loss of global vascular plant diversity by year 2050 as a result of climate change.

While species-area curves describe well-established empirical relationships, their use for estimating extinction rates has been the focus of debate. For example, He & Hubbell [11] suggested that species—area relationships always overestimate extinction rates derived from habitat loss; a number of other studies have similarly highlighted inadequacies in this approach [12, 13]. Extinction rates inferred from species-area curves have also been widely mis-interpreted [14]. As an alternative approach, Jetz et al. [15] used scenarios to evaluate the exposure of 8,750 terrestrial bird species to projected land-cover and climate change. Results indicated that at least 400 species are projected to experience >50% range reductions by the year 2050, and over 900 species by the year 2100. These range reductions were used to evaluate extinction risk by applying the IUCN Red List criteria, which indicated that the total number of threatened species in the analysis would increase by 19–30% by 2050 [15].

The IUCN Red List is widely recognised to be an authoritative approach for assessing the extinction risk of species, based on assessment of population sizes and population decline rates, and the extent and decline of geographic ranges [16, 17, 18]. This approach potentially offers a more robust method for assessing the impact of climate change on extinction risk. A number of studies have applied the Red List in this context. For example, Thuiller et al. [19] assessed 1,350 European plant species under seven climate change scenarios using Red List criteria, and found that more than half of the species could be threatened with extinction by 2080. Similarly, Bomhard et al. [20] examined 227 Proteaceae taxa endemic to the Cape Floristic Region, South Africa, under eight different land use and climate change scenarios for the year 2020. Overall, climate change was found to be a more significant threat than land use. Furthermore, Shoo et al. [21] used the Red List criteria to evaluate the impact of climate change on Australian rainforest birds, with 74% of species predicted to become threatened as a result of projected warming within the next 100 years.

However, in reference to such studies, Akçakaya et al. [22] suggested that the Red List criteria had often been misapplied, resulting in the introduction of substantial bias and uncertainty. Misapplications involved quantitative estimates of extinction risk, and inappropriate use of timescales, spatial scales, and spatial resolution. For example, the study by Thomas et al. [8] compared projected species distributions against the Red List thresholds for Area Of Occupancy (AOO) (i.e. the area occupied by a taxon), but failed to estimate AOO according to how it is defined in the Red List criteria. Similarly, the spatial resolution of data employed by Thuiller et al. [19] (i.e. 50 km x 50 km) was identified as being too coarse for assessing the status of individual species [22]. As a result of such methodological issues, the impacts of future climate change on biodiversity remain uncertain [2, 4, 7].

Here we examine the potential impact of climate change on extinction risk of tree species in the upper montane tropical Andes. This region was selected for study as tropical montane ecosystems are believed to be particularly at risk of climate change, as the potential for species to respond through migration is limited [1]. The region is also recognised as a global conservation priority, reflecting its high species richness and endemism [23, 24, 25, 26]. Few previous studies have examined the potential impacts of climate change on extinction risk of tree species, despite their high ecological and functional importance in many terrestrial ecosystems [27]. Here, analyses of climate change impact were conducted following a formal Red List assessment of these taxa [28], to enable a comparison between the impacts of climate change and other current threats. In this way, the research attempted to provide a robust evaluation of the relative effects of climate change on the extinction risk of species, in a region that is considered to be particularly vulnerable to such impacts.

## Materials and Methods

### Study area

The assessment focused on montane areas in the tropical Andes region above 1500 m a.s.l., occurring in Argentina, Bolivia, Colombia, Ecuador, Peru and Venezuela. Montane forests in this region extend from Venezuela, in the depression of Barquisimeto in Lara state, reaching their southernmost limit in northwest Argentina (29° S). The altitudinal limit of 1500 m was selected to ensure that all tree species restricted to upper montane forest were included in the assessment [28]. Species distributions were examined in an area covering Longitude -81.3° to -55.3° W and Latitude 12.3° N to -29.3° S.

### Formal Red List assessment

All of the selected taxa were first subjected to a formal Red List assessment, involving a network of regional specialists, and applying all Red List criteria, as described by Tejedor Garavito et al. [28]. This assessment focused only on taxa that are endemic to the region and are relatively broadly distributed (i.e. are present in more than one country), to avoid the potential confounding effects of threats other than climate change affecting narrow endemics. The assessment therefore applied to the entire global distribution of the selected species. A list of candidate species was created by accessing data from a range of sources, including the Missouri Botanical Gardens database (www.Tropicos.org), regional herbaria (Colombian National Herbarium (COL), Venezuelan National Herbarium (VEN), Bolivian National Herbarium (LPB), Herbarium of the Universidad Pontificia Católica in Ecuador (QCA) and San Marcos Herbarium of the Universidad Nacional Mayor de San Marcos, Peru (USM)), as well as regional floras and personal databases. The nomenclature of taxa on the list was checked using the Plant List (www.theplantlist.org, accessed March 2011), to identify synonyms and those species unresolved taxonomically. The Angiosperm Phylogeny Group III system was followed to provide taxonomic consistency.

Geographical distribution data for all of the tree species were then compiled. Sources included personal records of the network of regional specialists involved in the Red List assessment, the Missouri Botanical Gardens database (www.Tropicos.org), regional herbaria, and the Global Biodiversity Information Facility (GBIF: www.gbif.org), accessed during November 2010. A spatial database incorporating these distribution data was created in ArcGIS v.10 (ESRI, Redlands, California), then critically examined to exclude data points that were incorrectly georeferenced. The database was used to identify those species occurring exclusively at ≥ 1500 m a.s.l. by overlaying data on a Digital Elevation Model (DEM) obtained from www.worldclim.org, with a grid space of 30 arc seconds (0.0083° or approximately 1 km). If at least one of the records was below this altitudinal threshold, the species was excluded from further analysis. These records were also used to identify those species shared by more than one country. Distribution maps of each taxon that met these selection criteria were then checked by the regional network of specialists, and revised further where necessary.

The IUCN Red List criteria were then applied to each taxon, with reference to the distribution maps created [28]. In each case, the criteria were applied following the IUCN Red List guidelines [29, 30].

### Species distribution modelling

Assessing the potential effects of climate change on species distributions requires some form of modelling. As a number of different methods have been developed for modelling species distributions [31], a range of different methods were employed here, following Golicher et al. [32]. These were generalized additive models (GAM) [33], recursive partitioning (rpart) [34] and support vector machines (KSVM) [35], using a customised script in R statistical environment [36]. KSVM models were used from the ‘kernlab’ package, regression trees from the package ‘rpart’ and GAM from the ‘mgcv’ package, all of which are available in R. All the maps and outputs were resampled to a resolution of ~25 km^2^. Ideally, distribution models should include absence as well as presence data [37, 38], but as in this study, such data are rarely available at the scale of entire species ranges. Therefore 1000 randomly selected background data points were obtained to characterize the environment in the study region, following [32].

Current climate data were derived from the WorldClim data set [39], which is available at 1 km resolution. Following analysis of co-linearity using principal component analysis (PCA), four climate variables were selected based on PCA weightings and biological interpretability, following [32]. These were: 1) mean annual temperature, 2) mean diurnal range (mean of monthly (maximum temperature—minimum temperature)), 3) precipitation of the wettest month, and 4) precipitation of the driest month. These variables have been used previously to investigate the potential impact of climate change in the region [40,41] and in other neotropical montane forests [42].

For analysis of potential distribution under climate change scenarios, climate data were derived from the Hadley Centre Coupled Model for two of the Special Report on Emission Scenarios (SRES) for the year 2080, namely HADCM3 for scenario A2 and B2, following [32]. These were prepared for the Intergovernmental Panel on Climate Change (IPCC) Fourth Assessment Report [43] and were downscaled by Ramirez and Jarvis [44]. The A2 scenario projects a 3°C increase in surface air temperature by 2100 on average [45], whereas the B2 scenario projects an average 2.2°C temperature increase across all models [46].

Model validation was performed by splitting the data into two subsets using the median latitude as a splitting point. One of the subsets was used to build the distribution models and the other was used to test model predictions. These tests were carried for KSVM, Rpart and GAM models, using two different types of background points, the first collected from throughout the region (i.e. South America) and the second collected within the minimum convex polygon (MCP) of the species distributions [29,30]. This provides an overview of the potential discrimination properties of the models, taking into consideration the extent of the prediction area. The area under the receiver operating characteristic (ROC) curve, known as the AUC, was calculated to evaluate the performance of the models. ROC curves were calculated to measure the power of discrimination of the models for a subset of those species with the largest number of records (N>19). The AUC values were used to produce binary maps for calculation of the potential distributional area of the species. The AUC threshold was set as 0.9, consistent with a high degree of accuracy [47].

### Extent of Occurrence

The Extent of Occurrence (EOO) represents the area contained within the shortest imaginary boundary encompassing all the occurrence sites of a taxon [30]. Following IUCN Red List guidelines [29], EOO was estimated using an MCP calculated for each individual species, using R [36]. The IUCN Red List guidelines [29] also indicate that maps of potential habitat, which can be derived from remote sensing imagery, can be used to inform EOO estimates. For this purpose, a classified global land cover map for 2009 (referred to henceforth as ‘GlobCover’) produced by Arino et al. [48] was used to exclude non-forest areas with very low likelihood of tree species presence. These data were obtained from the MERIS imaging spectrometer, at a resolution of 300 m, which was reclassified to ~5 km.

### Red List assessment of climate change impacts

To assess the potential impacts of future climate change on extinction risk, IUCN Red List Criterion A3 [30] was used, as this explicitly considers the population size reduction that is projected, inferred or suspected to be met in the future. According to IUCN guidelines, population size reduction must be measured over the longer of either 10 years or three generations, up to a total of 100 years [29]. Here, an estimate of 70 years for three generations was used, together with climate change projections for the years 2080–2100. Criterion A3 was applied by estimating the decline in EOO resulting from climate change, from which a suspected population size reduction was inferred. This was achieved by comparing the EOO values projected under the climate change scenarios from the current EOO, using GAM outputs. In addition, for calculation of both current and projected EOO values, areas of unsuitable habitat were excluded using the GlobCover map. The IUCN Red List guidelines [29] highlight the need for caution when extrapolating model results under future climate scenarios beyond the range of data used to build the model. For this reason, EOO values projected under the climate change scenarios were also calculated for the current distribution areas of each species, by clipping projected distributions with the current MCP. This provides a relatively precautionary approach to assessing extinction risk, as it assumes there is no scope for species migration beyond current geographical range boundaries. Criterion A3 was applied to classify the extinction risk of species following the thresholds defined by IUCN [30] in terms of species population size decline, with values of ≥ 30%, ≥ 50% and ≥ 80% for Vulnerable (VU), Endangered (EN) and Critically Endangered (CR) categories respectively.

### Red List Index

The Red List Index (RLI) provides a method of tracking changes in extinction risk in a set of species, based on the proportion of species in each category on the Red List [49]. To evaluate the relative impact of climate change on the extinction risk of the tree species considered here, the RLI was calculated for (a) the results of the formal Red List assessment, (b) application of criterion A3 in isolation according to the two climate change scenarios, and (c) application of criterion A3 together with all other criteria considered in the formal Red List assessment.

The method of calculating RLI followed Butchart et al. [49], according to the formula:

$$RLI_{t}=\left(M-T_{t}\right)/M$$

where *M* is the number of species multiplied by the maximum category weight (*W*_EX_, which is the weight assigned to extinct species, namely 5), and *T*_*t*_ is the sum of the category weights multiplied by the number of species in each category (with weights of Critically Endangered = 4, Endangered = 3, Vulnerable = 2, Near threatened = 1, Least Concern = 0). Therefore, the maximum possible value of *T* is *M*, and RLI values can range from 0 (all species are Extinct) to 1 (all species are Least Concern) [49].

### Protected areas

The coverage of the regional network of protected areas in relation to upper montane tree species was examined by overlaying current and projected species distributions on a map of protected areas. The latter was downloaded from the World Database on Protected Areas [50] and incorporated in the ArcGIS database.

## Results

### Species selection and assessment

3750 tree species were initially identified as occurring in the upper montane forests of the Andes. From this total, 917 species were excluded as no georeferenced records were found at the time of the search. Another further 1287 species were excluded as all of their records fell entirely within the boundaries of a single country, and the species was not believed to be present in another country of the study area by the network of specialists. 1400 species had at least one record that occurred below the 1500 m altitude threshold and were therefore excluded from subsequent analyses. Of the 146 species remaining, a further 17 were excluded during checking, as a result of taxonomic revision. As a result, 129 taxa were evaluated according to the Red List criteria.

Distribution records for these taxa were filtered to identify unique records for each location. The total number of records for all the species was 1666, with *Cestrum peruvianum* having the largest number of records (65). 25 species had fewer than 5 records and were therefore excluded from the modelling analysis (following [51]).

### Model validation

The three different models fitted for the 13 species with the largest number of distribution records, when tested with background data from the whole region, showed high mean AUC values when averaged across all species: KSMV, 0.88 (SE ± 0.03); GAM, 0.82 (± 0.03); Rpart, 0.86 (± 0.02). Similar results were obtained from the three modelling methods; none of the models for any of the species were associated with an AUC value lower than 0.62 (Table 1). However, mean AUC values were lower for many species when the background points were selected from within the MCP around the current distribution of the species, namely KSMV, 0.62 (±0.05); GAM, 0.70 (±0.03); Rpart, 0.60 (±0.04) (Table 1). For five species, in this latter case, the AUC value was ≤ 0.4 with at least one of the methods. This shows that when values for model validation were restricted to locations within the current area of distribution of the species, the models’ ability to discriminate species presence against background data was reduced. Overall, GAM produced the highest mean AUC values and therefore the results from this model were used for subsequent analyses.

**Table 1:** AUC values for 13 of the most abundant tree species, based on number of distribution records, using four climatic variables for three modelling approaches (KSMV, Rpart, GAM).

Species	(a) All region			(b) Within MCP		
	Ksvm	GAM	Rpart	Ksvm	GAM	Rpart
*Aegiphila bogotensis*	0.88	0.87	0.92	0.75	0.79	0.57
*Aegiphila ferruginea*	0.94	0.62	0.90	0.35	0.68	0.47
*Aphelandra acanthus*	0.83	0.98	0.93	0.50	0.53	0.35
*Baccharis latifolia*	0.97	0.76	0.89	0.86	0.68	0.81
*Buddleja pichinchensis*	0.95	0.74	0.91	0.39	0.65	0.56
*Ceroxylon parvifrons*	0.86	0.69	0.70	0.59	0.68	0.70
*Cervantesia tomentosa*	0.86	0.83	0.81	0.93	0.90	0.89
*Cestrum peruvianum*	0.94	0.85	0.84	0.41	0.66	0.62
*Clusia sphaerocarpa*	0.95	0.98	0.92	0.81	0.89	0.75
*Escallonia resinosa*	0.90	0.88	0.86	0.59	0.53	0.40
*Geissanthus bogotensis*	0.99	0.89	0.89	0.76	0.67	0.53
*Hesperomeles cuneata*	0.72	0.78	0.80	0.54	0.73	0.57
*Ilex scopulorum*	0.66	0.82	0.83	0.62	0.70	0.60

Models were validated using background data (a) from the entire region, and (b) from within the minimum convex polygon (MCP) of the current distribution of each species.

### Predictions of current distribution

**Fig 1:**
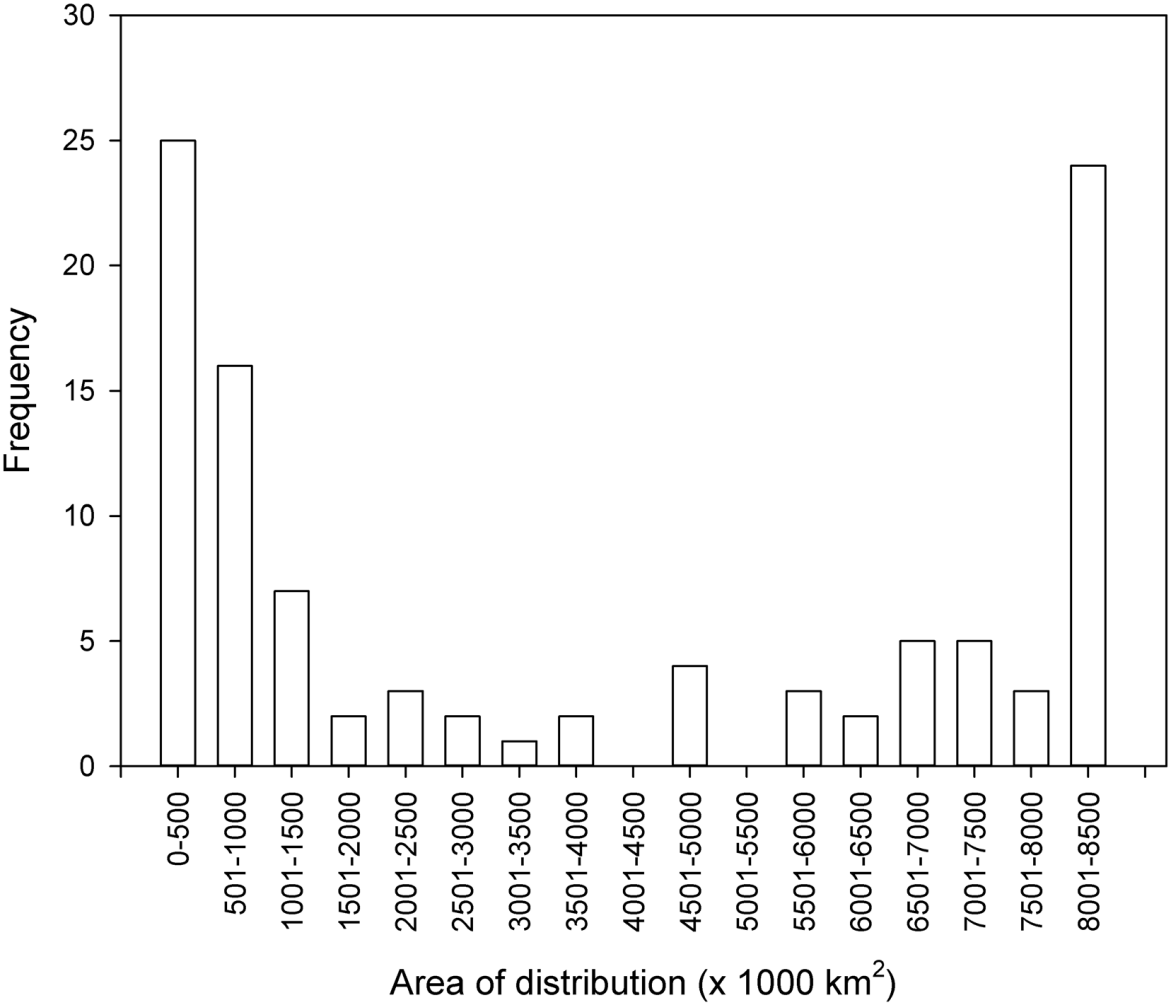
Frequency distribution of the area of species' potential distribution from the model (GAM) projections.

Using the results of the GAM model, the potential distributional area of many species extended broadly over the region, with a mean (± SE) area of 3,767,049 (± 326,364) km^2^. Values ranged from 114,525 km^2^ for *Calliandra taxifolia* to a maximum of 8,069,350 km^2^ for 14 species, namely: *Axinaea grandifolia*, *Azara salicifolia*, *Cinchona pyrifolia*, *Clusia pseudomangle*, *Clusia sphaerocarpa*, *Cyathea catacampta*, *Cybianthus laetus*, *Gynoxys calyculisolvens*, *Gynoxys sancti-antonii*, *Oreopanax seemannianus*, *Schefflera inambarica*, *Solanum goniocaulon*, *Weinmannia auriculata*, *Zanthoxylum brisasanum*. Overall, the frequency distribution for modelled potential distribution was bimodal, with peaks in the 0–500 and 8001–8500 x 1000 km^2^ categories, but with relatively low values in most of the other categories (Fig 1).

As expected, restricting the species’ distributions by subtracting non-suitable habitat reduced the potential distribution areas considerably, resulting in an overall mean area of 451,234 (± 20,710) km^2^. Values ranged from a minimum of 53,825 km^2^ for *Ilex rimbachii* to a maximum area of 657,375 km^2^ for 23 species, namely the same 14 species listed above with the addition of *Daphnopsis espinosae*, *Dendrophorbium balsapampae*, *Ilex scopulorum*, *Ilex uniflora*, *Miconia harlingii*, *Nectandra subbullata*, *Persea brevipes*, *Prunus urotaenia* and *Ribes canescens*.

**Fig 2:**
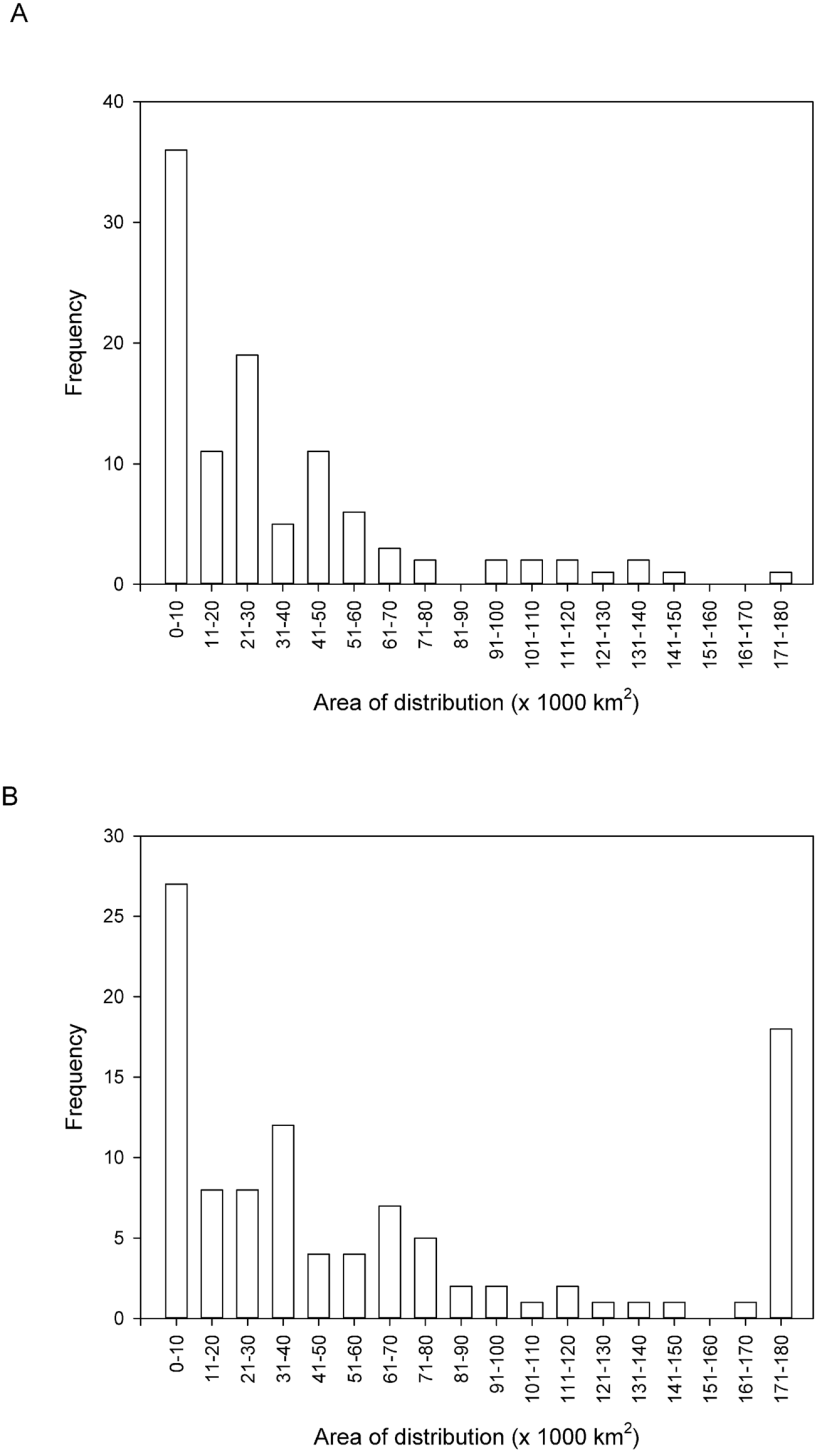
Frequency of species’ potential distribution derived from the model (GAM) projections, restricted by clipping with the MCP drawn around current distribution data, and (a) excluding unsuitable habitat (i.e. by using the ‘GlobCover’ map), (b) not excluding unsuitable habitat.

The estimates of distributional range were further restricted by clipping them with the MCP around current distribution data, to enable comparison between model outputs and current patterns of distribution. In this case, the mean projected area was 32,965 (± 3,569) km^2^ (Fig 2). Area values for 47 species were <20,000 km^2^, which is the EOO threshold for the threatened categories under the B1 criteria [30].

### Prediction of potential distribution under climate change scenarios

When EOO values were calculated without excluding unsuitable areas, the results for the GAM model showed that for 10 species, the projected area under the A2 climate change scenario was larger than the current potential distribution. These species were: *Citharexylum joergensenii*, *Clethra rugosa*, *Cyathea frigida*, *Geissanthus argutus*, *Ilex uniflora*, *Prunus pleiantha*, *Schinus pearcei*, *Schoepfia flexuosa*, *Senna versicolor* and *Smallanthus fruticosus*. For the other 94 species the projected area under climate change was less than the current potential distribution. Under the B2 scenario, 12 species had a larger projected distribution than their current potential distribution, namely: *Berberis lehmannii* and *Dendrophorbium balsapampae*, and the 10 species listed under the A2 scenario. For the other 92 species, the projected distributional area was less than the current potential distribution.

**Fig 3:**
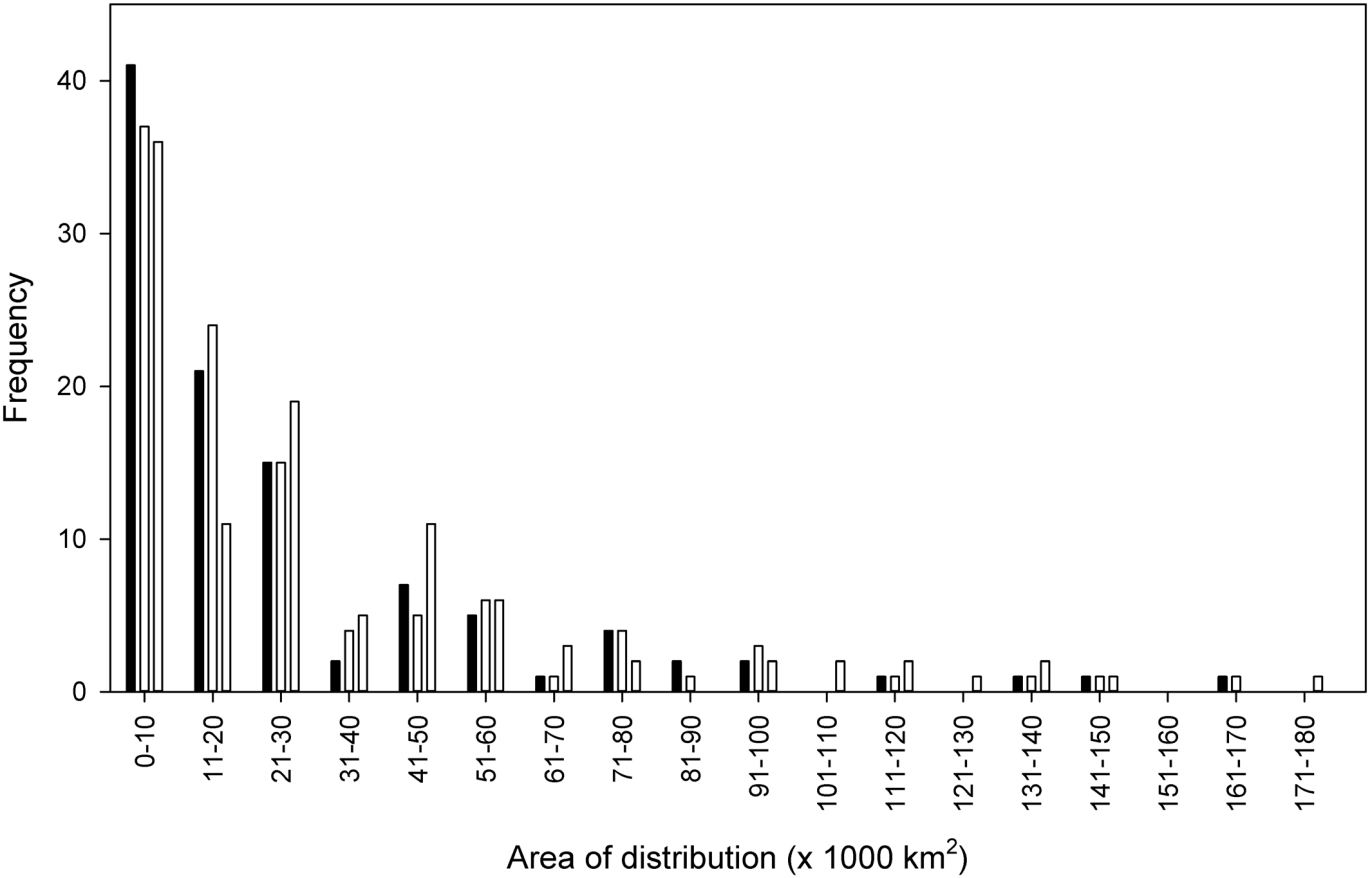
Frequency of species’ potential distribution derived from the model (GAM) projections under climate change scenarios A2 (black) and B2 (grey), and current potential distribution (white), excluding unsuitable habitat (i.e. by using the ‘GlobCover’ map), and restricting the range to the MCP drawn around current distribution data.

As expected, when EOO values were calculated by excluding unsuitable areas and by clipping with the MCP around current distribution data, the projected species distributions under the climate change scenarios were considerably reduced. Using these lower EOO values, under the A2 scenario the projected distributional area decreased for 73 species and remained unchanged for 27 species. Under the B2 scenario, the projected area decreased for 75 species and remained unchanged for 25 species. The mean projected distributional area for the species under the A2 scenario was 26,198 (± 3,193) km^2^ and for the B2 scenario was 27,124 (± 3,208) km^2^. The lowest values were recorded for *Alchornea anamariae*, for which projected area was reduced from 3,250 km^2^ to 0 km^2^, and *Berberis jobii*, for which values were 0 km^2^ under both scenarios. The mean difference between the area of current potential distribution and the area projected to be suitable for each species under climate change (within the MCP) was 7,177 (± 1,088) km^2^ (28%) and 5841 (± 947) km^2^ (23%), for the A2 and B2 scenarios respectively (Fig 3).

### Impact of climate change on Red List classification

When the projected distributional area of each species was used, resulting from the GAM, none of the species met any of the EOO thresholds associated with threatened Red List categories. In this case, all species were classified as LC. When currently unsuitable areas were subtracted from the EOO estimate using the “Globcover” map, all species again classified as LC. When estimates of EOO were further reduced by clipping with the MCP associated with current distributional range, 46 species qualified as threatened according to the A3 criterion, under the A2 climate change scenario. These included 9 species meeting the threshold for CR, 10 species for EN and 27 species for the VU threshold (Table 2). A slightly lower total of 36 species qualified as threatened under the B2 scenario, according to criterion A3 (Table 2).

**Table 2:** Number of tree species threatened with extinction according to the IUCN Red List categories and criteria.

Category	RL current threats (a)	Present (b)	Model present (c)	A2 (d)	B2 (e)	Highest (f)
CR	1	0	1	9	4	8
EN	47	17	20	10	10	63
VU	28	31	26	27	22	34
NT	19	0	0	0	0	5
LC	29	72	57	58	68	16
DD	5	9	25	25	25	3

^(a)^ Results of the Red List assessment conducted with all Red List criteria, based on a consideration of current threats, but excluding the potential impacts of climate change (from Tejedor Garavito et al. [28]).

^(b)^ Number of species in each Red List category meeting the thresholds associated with criterion B1 only, using an estimate of EOO based on current distribution data, with area of unsuitable habitat subtracted (using Globcover map and altitudinal threshold).

^(c)^ Number of species in each Red List category meeting the thresholds associated with criterion B1 only, using an estimate of EOO based on modelled potential present distribution using current climatic variables, with area of unsuitable habitat subtracted (using Globcover map and altitudinal threshold), and further limited to the minimum convex polygon based on current distribution.

^(d)^ Number of species in each Red List category meeting the thresholds associated with criterion A3 only, using an estimate of EOO based on modelled potential distribution using climatic variables from the A2 scenario, with area of unsuitable habitat subtracted (using Globcover map and altitudinal threshold), and further limited to the minimum convex polygon based on current distribution.

^(e)^ Number of species in each Red List category meeting the thresholds associated with criterion A3 only, using an estimate of EOO based on modelled potential distribution using climatic variables from the B2 scenario, with area of unsuitable habitat subtracted (using Globcover map and altitudinal threshold), and further limited to the minimum convex polygon based on current distribution.

^(f)^ Number of species in each Red List category, considering both current threats and potential future climate change, and based on application of all Red List criteria. Abbreviations: CR, Critically Endangered; EN, Endangered; VU, Vulnerable; NT, Near Threatened; LC, Least Concern; DD, Data Deficient.

Note that the number of species classified as DD varied between columns according to the method used. For the first column, species were classified as DD according to the expert opinion of the network of specialists; for the second column, those listed as DD were those with fewer than three distributional points (required for MCP), and for those columns involving GAM, those species with fewer than five records were excluded from the modelling and classified as DD.

Comparing these results with those of the formal Red List assessment indicated that under the A2 scenario, 26 species would be uplisted in the category of threat, with five species moving from VU to CR, five from LC to VU, two from LC to EN and one from LC to CR (Table 3). Under the B2 scenario, 23 species would be uplisted in threat category, with a total of four species moving to CR from NT, VU and EN, eight species moving to EN from VU, NT and LC, and 11 to VU from NT and LC (Table 4).

**Table 3:** Matrix to illustrate transition between Red List categories as a result of climate change, according to the A2 climate change scenario.

Red List assessment							
Category	CR	EN	VU	NT	LC	DD	Total
CR		1	5	2	1		9
EN		4	2	2	2		10
VU		8	8	6	5		27
LC		21	12	7	17	1	58
DD	1	13	1	2	4	4	25
Total	1	47	28	19	29	5	129

The columns present the number of species in each Red List category as a identified in the formal Red List assessment (Tejedor Garavito et al. [28]). Rows indicate the number of species in each Red List category according to application of criterion A3 only, relating explicitly to the impact of climate change. Application of criterion A3 was based on the projected change in EOO resulting from climate change, in which currently unsuitable habitat was excluded (by subtracting non-forest areas in the ‘Globcover’ map and areas projected as unsuitable using the GAM), within the current distributional range of the species. Abbreviations: CR, Critically Endangered; EN, Endangered; VU, Vulnerable; NT, Near Threatened; LC, Least Concern; DD, Data Deficient.

**Table 4:** Matrix to illustrate transition between Red List categories as a result of climate change, according to the B2 climate change scenario. For details, see caption to Table 3.

Red List assessment							
Category	CR	EN	VU	NT	LC	DD	Total
CR		1	2	1			4
EN		2	4	2	2		10
VU		5	6	6	5		22
LC		26	15	8	18	1	68
DD	1	13	1	2	4	4	25
Total	1	47	28	19	29	5	129

### Red List Index (RLI)

The overall value of the RLI for the formal Red List assessment [28] was 0.66. When criterion A3 was applied in isolation to evaluate the impact of climate change on extinction risk, RLI values were 0.74 and 0.78 for the A2 and B2 climate change scenarios respectively. When criterion A3 was applied in conjunction with the other Red List criteria, as employed in the formal assessment, the RLI values were 0.55 and 0.57 for the A2 and B2 climate change scenarios respectively. When the highest threat category from either climate change scenario was applied, an RLI value of 0.56 was obtained. This represents a 15% decline in the RLI value compared to that of the formal Red List assessment, which provides a measure of the relative impact of climate change on extinction risk compared to other current threats.

### Coverage of protected areas

**Fig 4:**
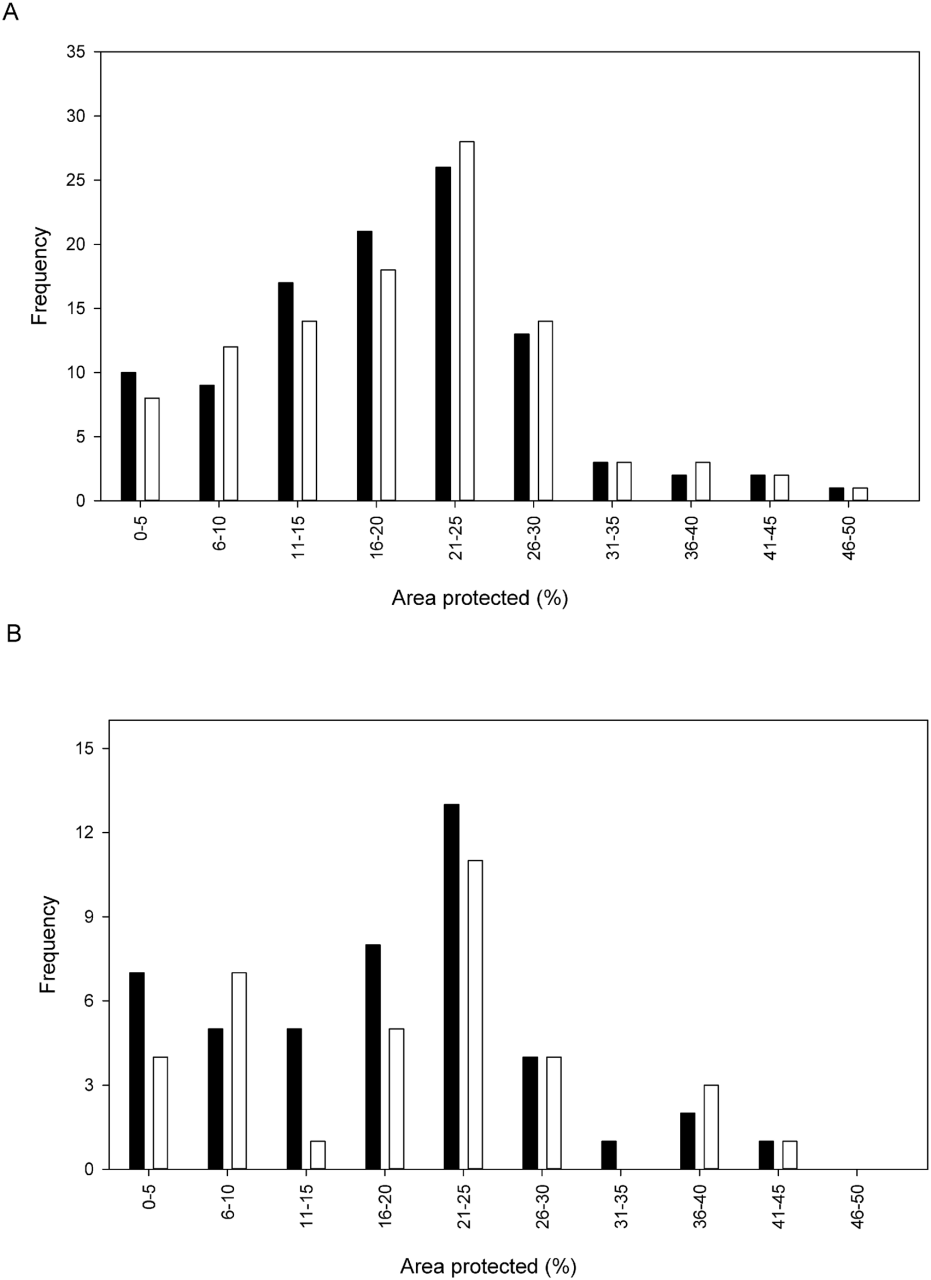
Frequency distribution of the percentage of EOO that is located within a protected area a) for all of the species, using modelled potential distribution, and b) for those species classified as threatened, according to Red List criterion A3, for climate change scenarios A2 (black) and B2 (white).

The total area of tropical montane forest >1500 m a.s.l. designated as protected within the study area was ~110,625 km^2^. Based on analysis of the modelled current potential distribution of each species, the mean percentage this area that was located within a protected area was 19.6 (± 0.91)%. The potential distributions of two species, namely *Berberis jobii* and *Cyathea catacampta*, were found to lie entirely outwith protected areas, while *Citharexylum joergensenii* was associated with the highest percentage area protected, with a value of 50%. Under the A2 and B2 climate change scenarios, the mean percentages of EOO that were located within a protected area were slightly lower, with values of 18.6 (± 0.97)% and 19.1 (± 0.96)% respectively (Fig 4). Under scenario A2 seven species had no protected areas located within their EOO, namely *Alchornea anamariae*, *Berberis jobii*, *Calliandra taxifolia*, *Cyathea catacampta*, *Palicourea candida*, *Prunus pleiantha*, *Symplocos canescens*. Under scenario B2, four species were entirely located outwith protected areas, namely *Alchornea anamariae*, *Berberis jobii*, *Cyathea catacampta* and *Symplocos canescens*. For those species that were considered to be threatened according to criterion A3, taking into consideration the projected distribution under the climate change scenarios, the mean percentage areas that were protected were 18.3 (± 0.97)% and 18.7 (± 0.95)%, for A2 and B2 scenarios respectively.

## Discussion

The current results indicated that climate change increased the risk of extinction of 18–20% tree species evaluated, depending on the climate scenario. The overall impact is illustrated by the 15% decline recorded in the value of the Red List Index, when climate change was considered in addition to other current threats. In other words, climate change accounted for a 15% increase in the overall risk of extinction, in addition to current threats, according to values of this index. While these results suggest that climate change represents a significant threat to tree species in the tropical Andes, they contradict suggestions that climate change will become the most important cause of biodiversity loss in coming decades [2, 4, 7, 8, 20, 52, 53]. However, they support the results obtained by Jetz et al. [15], who found for birds that habitat loss in tropical countries will likely pose a more immediate threat to a greater number of species than will climate change.

This research was explicitly designed to assess the relative impact of climate change on a group of species that are likely to be particularly vulnerable to this threat. High-elevation specialist species in the tropics are considered to be among the most vulnerable to extinction as a result of climate change [8, 52, 54, 55, 56, 57, 58]. Such species have limited capacity to respond through migration, because the area available for dispersal at high elevations is limited [15, 59]. Many tropical species are believed to be thermally specialised, with narrow elevational ranges, which further increases their vulnerability [60]. Climate change projections for the tropical Andes suggest that significant warming will occur in the 21st century, particularly at higher elevations, together with a significant increase in interannual temperature variability [61]. Changes in precipitation are also forecast, with both regions of increased and decreased precipitation projected in different Andean locations [61]. Climate change could also affect other key features of tropical montane environments, such as the height of the cloud base and moisture inputs from cloud-stripping, with consequent impacts on species [59]. Furthermore, climate change could lead to expansion of the agricultural frontier to higher elevations, which could contribute to the further fragmentation and loss of forests [62].

The current results are partly attributable to the fact that upper montane species are currently being subjected to a number of existing threats. Pre-eminent among these is forest loss and degradation, caused by conversion of forest to agricultural land use, and over-exploitation of tree species for timber and fuelwood. Estimates of deforestation rate vary from 0.17–1.89% per annum, depending on the country [63], with a regional mean value of 0.62% per annum in recent decades [64]. Tree species of high socio-economic value such as *Cinchona* spp., *Podocarpus* spp., *Zanthoxylum* spp., *Polylepis* spp. and *Ilex* spp. continue to be subjected to overexploitation [28]. Other threats affecting tree species in the region include fire, browsing animals, urban expansion, infrastructural development and mining [28]. Climate change therefore represents an additional potential threat to species that are currently being subjected to multiple existing threats. Climate change might also interact with other threats such as spread of pests and diseases and fire [65], although such interactions were not explicitly considered here.

The current results should clearly be interpreted with caution, as they are based on a number of assumptions. First, the distribution of tropical montane tree species is imperfectly known, and it is therefore possible that the geographical ranges were underestimated. The lack of distribution data, commonly referred to as the Wallacean shortfall [66], is a widely recognised problem in conservation biogeography in tropical regions [67], and limits the precision of conservation assessments such as those reported here. The projections of climate change employed here should also be considered as uncertain, as are all such climate scenarios [68]. It should be noted that since this research was conducted, the climate scenarios developed by the IPCC have been revised further (AR5), which should also be borne in mind when interpreting the results.

A further major area of uncertainty relates to the use of species distribution models to evaluate climate change impacts. While environmental niche modelling approaches are widely believed to offer the best available tool for rapid assessments of potential range changes [2, 69], a number of limitations have been identified [13, 70, 71]. First among these is the amount and quality of available data, which are often partial and biased [47, 67]. The presence data used here may have been biased, as well as incomplete, as they were not based on the use of a systematic and comprehensive sampling strategy [72]. Species distribution modelling approaches fail to consider the role of factors such as species dispersal capabilities, life history characteristics and interactions between species in determining the ability of species to track climate change [73]. As a result, species distributions may not meet the assumption of equilibrium with climatic variables on which niche-based models are based [69]. This is particularly the case in human modified landscapes such as those examined here, where the ability of species to migrate in response to changing climate could be limited by anthropogenic impacts such as forest fragmentation.

A further issue is the use of artificial absence data (i.e. pseudo-absences or background data) in species distribution models. While this approach is widely implemented because of the difficulties of obtaining confirmed absence data, it can have negative effects on model performance [74]. For this reason, two different approaches to selecting pseudo-absence data were applied here, with lower AUC values obtained when pseudo-absences were restricted to locations within the current area of distribution of the species. Again, this uncertainty should be borne in mind when interpreting the results. A further source of uncertainty relates to potential variation between different modelling methods, although here similar results were obtained here regardless of the method used, in common with some previous investigations [32, 75].

The IUCN Red List assessment process is also associated with a number of uncertainties [30]. Key assumptions in the current analysis included: (i) species distribution models were equally valid for all species, although robust validation was only possible for the subset of species with a relatively large number of distributional records; (ii) the Globcover map of forest cover provides an accurate indication of suitable habitat for tree species, noting that this does not take into account any potential future land cover change in the region; (iii) species abundance was linearly related to EOO estimates, as applied in criterion A3. Further, it should be noted that local or national endemic species were excluded from the current analysis, reflecting the fact that the distributional ranges of such species are less likely to be in equilibrium with current climate, as assumed by species distribution modelling approaches. Some 467 local endemics have been assessed by previous national Red List assessments, of which 165 (35%) were classified as threatened [28]. Potentially, some of these species could also be threatened by climate change, but this is difficult to assess accurately using niche modelling approaches.

In addition, clipping the maps of potential distribution by the MCP of current distribution provides a relatively precautionary approach, as it assumes there is no scope for species migration beyond current geographical range boundaries in response to climate change. Results indicated that if species were able to occupy all areas of potential habitat under climate change scenarios, none would be threatened with extinction explicitly by climate change. Few data are available on how the distribution patterns of tree species might change in response to climate change, although Feeley et al. [76] recently recorded a mean vertical rate of migration of approximately 2.5–3.5 m per year for trees along an elevational gradient in Peru. As temperatures in the tropical Andes have increased at a rate of approximately 0.03–0.04°C year since 1975, a value that exceeds the global mean [77], a migration rate of 5.5–7.5 m year would be required to keep track of the current rate of climatic change.

Despite the limitations of the current research, the results indicate that climate change may have implications for the development of conservation strategies in the region. Under the scenarios examined here, model projections suggest that climate change would increase the number of tree species that are not included within the current boundaries of protected areas. Similarly in Europe, Araújo et al. [78] found that 58% of species considered would lose suitable climate in protected areas. Such results highlight the importance of considering potential climate change impacts for informing conservation planning and management approaches at a regional scale [79]. Management options include increasing the size and number of protected areas, and the degree of connectivity between them; and site- or species-specific approaches such as managed translocation (assisted migration), habitat manipulations and ecosystem engineering [80]. The exploration of such options in the Andean region has been very limited to date. However, the current results highlight the importance of not focusing solely on climate change when developing such strategies; there is also an urgent need for strengthened conservation action to address the many existing threats currently affecting biodiversity in this globally important region.
